# The patterns of acetylcholinesterases during developmental stages of *Aedes aegypti* and their susceptibility toward insecticides in egg stage

**DOI:** 10.1038/s41598-026-45818-1

**Published:** 2026-04-18

**Authors:** Saleh A. Mohamed, Azza M. Abdel-Aty, Hasan A. Al-Talhi, Ahmed N. Abo-Khatwa, Khaled M. Al-Ghamdi

**Affiliations:** 1 Molecular Biology Department, National Research Center, Dokki, Cairo Egypt; 2 Biochemistry Department, Faculty of Science, King Abdulaziz University, Jeddah, 21589 Kingdom of Saudi Arabia; 3 Biology Department, Faculty of Science, King Abdulaziz University, Jeddah, 21589 Kingdom of Saudi Arabia

**Keywords:** *Aedes aegypti*, Developmental stages, Acetylcholinesterase, Organophosphates, Carbamates, Biochemistry, Chemical biology, Zoology

## Abstract

The screening of acetylcholinesterase (AChE) during developmental stages, eggs, larvae, pupa and adults of *Aedes aegypti* was detected. Chromatography on a Sephacryl S-200 column revealed two distinct peaks of acetylcholinesterases AChE1 and AChE2 in every developmental stage. The egg stage had the highest expression of AChE1 activity, which progressively declined throughout the larval, pupal, and adult stages. On the other hand, AChE2 activity was lowest in eggs and larvae, progressively increased in pupae, and peaked in adults. To avoid overlap with larval development, the properties of AChE1 and AChE2 from 36 h-old eggs were detected rather than 48 h-old eggs. The enzymes’ substrate specificity revealed that, in comparison to acetylthiocholine iodide (AcSChI), the relative activity percentage for butyrylthiocholine iodide was 9.4 for AChE1 and 11.6 for AChE2. *Ae. aegypti* AChE1 and AChE2 had Km values of 0.256 and 0.288 mM AcSChI, respectively. The optimal pH and temperature for both enzymes were 7.5 and 40˚C, respectively. Up to 40˚C and 25˚C, respectively, the activity of AChE1 and AChE2 were thermally stable. With the exception of Co^+ 2^, every metal tested had a partial inhibitory impact on the two enzymes. The most sensitive organophosphates and carbamates for inhibition of both enzymes were detected in eggs for the first time. For organophosphates and carbamates, the type of inhibition, bimolecular rate constant (Ki), and Ki ratio AChE1/AChE2 were detected. According to the findings, the Ki ratio AChE1/AChE2 toward methomyl, chlorpyrifos-methyl, and pirimiphos-methyl is less than 1.0, suggesting that both enzymes are more susceptible to inhibition by these insecticides. Eserine, malathion, and fenitrothion, on the other hand, have Ki ratios AChE1/AchE2 more than 1.0, indicating lower sensitivity. The study recommended using methomyl, pirimiphos-methyl, and chlorpyrifos-methyl to attack *Ae. aegypti* during the egg stage.

**Supplementary Information:**

The online version contains supplementary material available at 10.1038/s41598-026-45818-1.

## Introduction

*Aedes aegypti* is one of the main vectors of the vector-borne viral diseases dengue, zika, and chikungunya, which are spread by mosquitoes^1,2^. These diseases have become much more common in urban and semi-urban areas over the past few decades, which has created major problems for public health in terms of morbidity and mortality, especially in low-income nations where access to medical care is restricted^3,4^. Many strategies have been used to lower the rate at which these viruses spread, mostly by managing the A. *aegypti* population. Insecticides based on organochlorines, organophosphates, carbamates, or pyrethroids are used in the majority of these strategies^5^. Acetylcholinesterase enzyme (AChE) inhibition is one of the main ways insecticides work; chronic AChE inhibition suggests a breakdown in acetylcholine homeostasis, which can eventually cause paralysis and insect death^6^.

One important neurotransmitter in insect neural transmission is acetylcholine (ACh)^7^. A crucial molecular target for mosquito control strategies is AChE, which controls ACh levels at the synapse^8^. Choline acetyltransferase converts choline and acetyl-CoA into ACh. After transmitting brain signals in the synaptic cleft, it is quickly digested by AChE into choline and acetate. Presynaptic neurons then reabsorb these compounds for future usage. According to structural research, AChE’s catalytic effectiveness is enhanced by its small, deep active site that is bordered with aromatic amino acids^9^. Inhibition of AChE results in the accumulation of ACh in the synaptic cleft, causing overstimulation of nerve receptors. This leads to paralysis and ultimately death in insects^10,11^. Mosquitoes express two isoforms of AChE: AChE1 and AChE2, whereas humans and mammals usually express just one type. The most common and important isoform in mosquitoes among these is AChE1^12,13^. Insecticides that target AChE, such as organophosphates and carbamates, disrupt nerve transmission by inhibiting this enzyme, leading to neuromuscular overstimulation and insect mortality^11,14–16^. These chemicals’ specificity permits targeted control of vector populations and demonstrates a sophisticated understanding of mosquito neurology^17^.

Despite the growing interest in biological control methods, especially the use of pesticides produced from plants. These plant-based substances provide more sustainable and safe options for controlling mosquitoes. In contrast to synthetic pesticides, which frequently increase mosquito detoxification enzyme activity, phytochemicals produced from plants directly target vital cellular processes, impairing physiological activities^18–21^. As of right moment, synthetic insecticides are more effective than plant compounds at inhibiting AChE from *Ae Egypti*. Since AChE is mostly linked to synaptic neurotransmission and pesticide target-site interactions, the majority of prior research on insect AChE has concentrated on the larval and adult stages^22^. Prior to the development of a functional nervous system, acetylcholine primarily acts as a non-neuronal signaling molecule involved in embryogenesis, cell proliferation, and differentiation during the egg stage, a crucial but little-studied developmental window^23^. The current study closes a significant information gap about the embryonic control of cholinergic systems in insects by characterizing AChE activity and isoform distribution at this early period. Therefore, 36-hour egg stage was chosen to identify the most susceptible carbamates and organophosphates to inhibition of both enzymes. Thus, the objective of this research is to isolate AChE1 and AChE2 from every stage of *Ae Egypti* growth. For the first time, the 36-hour egg stage was chosen to identify the most susceptible carbamates and organophosphates to inhibition of both enzymes. Generally, AChE is primarily studied in larvae and adults of *Ae egypti* rather than in eggs. The physiochemical characterization of AChE1 and AChE2 were also investigated.

## Materials and methods

### Chemicals

Acetylthiocholine iodide (AcSChI), butyrylthiocholine iodide and eserine were purchased from Sigma. Sephacryl S-200 was obtained from Pharmacia Fine Chemicals. Malathion, methomyl, chlorpyrifos-methyl were obtained from National Research Centre, Cairo, Egypt. Fenitrothion and pirimiphos-methyl was obtained from the department of pest control, Jeddah municipality. Other chemicals were of analytical grade.

### *Aedes aegypti*

The 27th generation local strain of *Ae. aegypti* was obtained from the municipality’s Insect Lab., Department of Pest Control and Public Health of Jeddah Municipality, Jeddah, Saudi Arabia.

### *Aedes aegypti* rearing

Mosquito colonies were reared in a sterilized insectary room. The insectary room was maintained at 28 °C and ~ 80% humidity, with a 12 h day/night cycle. Larvae were fed on an artificial grounded fish food and adults were fed on a 10% sucrose solution. The eggs obtained from the municipality insect lab were placed in a plastic tray 125 cm^3^ filled with distilled water to a depth of 2.5 cm. The eggs were hatched within 2–3 days. The larvae took about 5–7 days before they reached pupal stage which was separated out from the remaining larvae into a cartoon cup filled with distilled water and placed inside a new labeled 30 cm^3^ cage until adult emergence. Adults were provided blood meals by restrained pigeons which placed on the top of the cloth-made cage for an hour. After 48 h, a filter paper Whatman No. 4 was fitted on a cartoon cup with ~ 75 ml distilled water and placed inside the cage. A batch of eggs were collected every 12 h and kept under the same conditions until the appropriate age (24, 36 and 48 h) and transferred to frozen storage. The other batch of eggs were hatched after 48 h to four developing instars larvae followed by pupa stage and collected individually and transferred to frozen storage. Adults were collected using a simple insect aspirator and transferred to frozen storage.

## Partial purification of acetylcholinesterase

### Preparation of crude extract

Six hundred mg of *Ae. aegypti* eggs, larvae, pupa and adults were separately homogenized in 10 ml of 20 mM sodium phosphate buffer, pH 7.5 and centrifuged at 10,000 rpm for 20 min at 4 °C. The supernatant was designated as crude extract and stored at −15 °C until used.

### Chromatography on Sephacryl S-200

The crude extracts of acetylcholinesterase from *Ae. aegypti* 36-h old eggs, larvae, pupa and adults were separately loaded on Sephacryl S-200 column (90 × 1.6 cm i.d.) previously equilibrated with 20 mM sodium phosphate buffer, pH 7.5 and developed at a flow rate of 30 ml/h and 2 ml fractions were collected. The enzyme was eluted with the same buffer.

### Acetylcholinesterase assay

A technique outlined by Ellman et al.^24^ was used to measure acetylcholinesterase activity. 20 mM sodium phosphate buffer, pH 7.5, 1 mM DTNB, 1 mM (AcSChI), and 0.1 unit of enzyme made up the standard reaction mixture, which had a final volume of 1.0 ml. The absorbance was measured at 412 nm after the reaction mixture was incubated for one hour at 37 °C. Under conventional test conditions, the specific activity is expressed as units/mg protein. One unit of acetylcholinesterase activity is defined as the quantity of enzyme that releases 1 µmol of thionitrobenzoic acid per hour. Thionitrobenzoic acid had a molar extension coefficient of 13,600 M^− 1^ cm^− 1^.

### Protein determination

Protein was quantified by the method of Bradford^25^ with bovine serum albumin as standard.

### K_m_

The K_m_ value was determined from Lineweaver-Burk plot by using AcSChI concentrations from 0.2 to 1.2 mM under standard assay conditions.

### pH optimum

AChE activity was determined at various pH using different buffers, sodium acetate (pH 5.5–6.0.5.0) and sodium phosphate buffer (6.5–8.8) at 20 mM concentration. The maximum activity was taken as 100% and % relative activity plotted against different pH values under standard assay conditions.

### Temperature optimum

AChE activity was determined at a temperature range of 10–70 °C. The maximum activity was taken as 100% and % relative activity were plotted against different temperatures under standard assay conditions.

### Thermal stability

The enzyme was incubated at a temperature range of 10–80 °C for 30 min prior to substrate addition. The % relative activity was plotted against different temperatures under standard assay conditions.

### Effect of metal ions

The enzyme was incubated with 1 mM solution of Mg^2+,^ Co^2+^, Ca^2+^, Cu^2+^, Ni^2+^, Zn^2+^ and Hg^2+^ for 15 min prior to substrate addition. The enzyme activity without metal ions was taken as 100% and % relative activity was determined in the presence of metal ions under standard assay conditions.

### Kinetics of insecticides

The kinetics of inhibition for enzymes was determined in the presence of 0.2–7.0.2.0 µM organophosphates, malathion, chlorpyrifos-methyl, fenitrothion and pirimiphos-methyl, and carbamates, eserine and methomyl. The Lineweaver-Burk plot can be used to determine K_i_ from the values of the appropriate intercepts in the presence and absence of inhibitor under standard assay conditions.

### Statistical analysis

The data were analyzed using a one-way ANOVA, followed by Tukey’s post hoc test and correlation analysis, all performed with GraphPad Prism 5 software. Results are presented as means ± standard deviation (*n* = 3), with statistical significance at *P* < 0.01.

All experimental procedures were carried out in compliance with relevant guidelines.

## Results and discussion

**Fig. 1:**
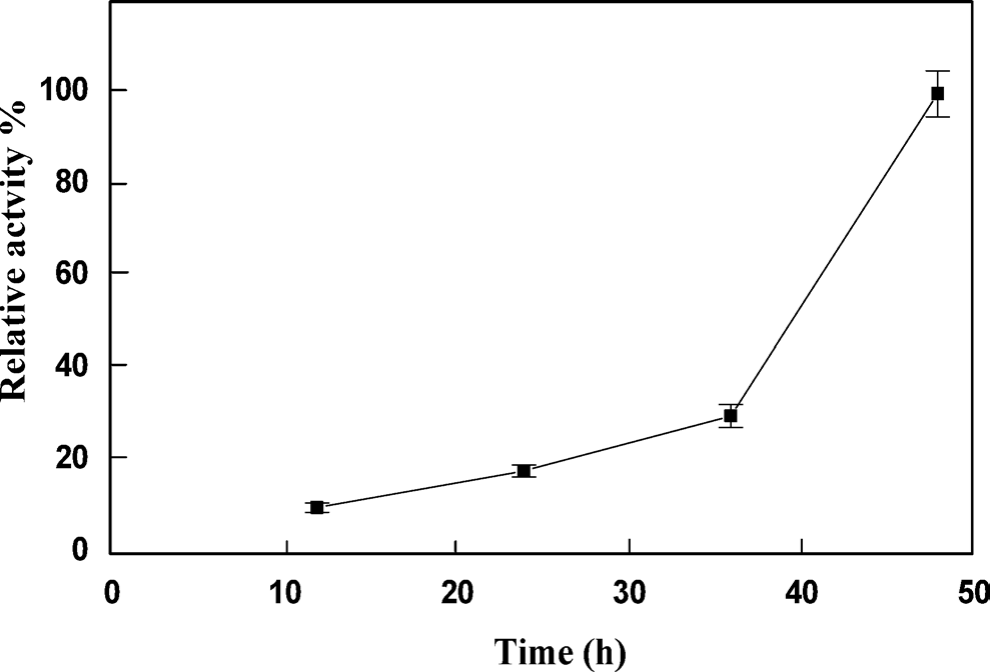
Changes in AChE activity of *A. aegypti* egg crude extracts during embryogenesis. Each point represents the mean of three runs for each developmental stage ± S.E.

**Fig. 2:**
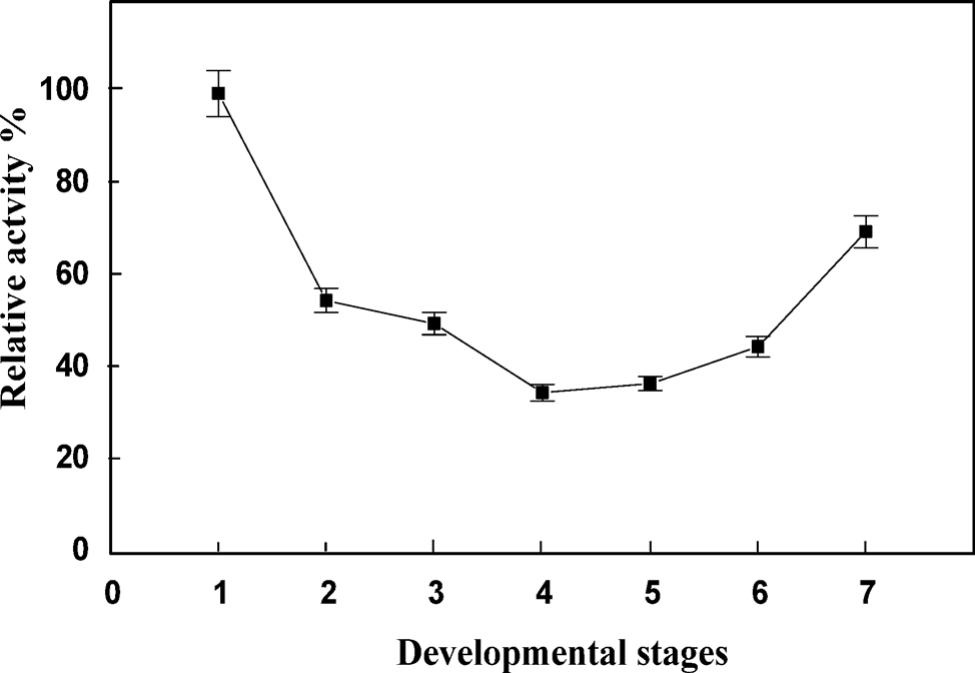
Changes in AChE activity during developmental stages of *Ae. aegypti* 1- egg, 2- first instar larvae, 3- second instar larvae, 4- third instar larvae, 5- fourth instar larvae, 6- pupa, 7- adult crude extracts. Each point represents the mean of three runs for each developmental stage ± S.E.

Very little information has been reported on the screening of AChE during developmental stages of *Ae. Aegypti*. Previously, AChE was detected in adults of *Ae. Aegypti*^26^. In the present work, the activity of AChE during development stages of *Ae. aegypti* was screened. The activity profile of AChE during egg stage showed the highest activity at 48 h after egg laying (Fig. 1). The activity started to diminish gradually from first-, second-, third- and fourth- instar larvae. The activity was gradually increased at pupa stage and reached its high activity at adult stage (Fig. 2).

**Fig. 3:**
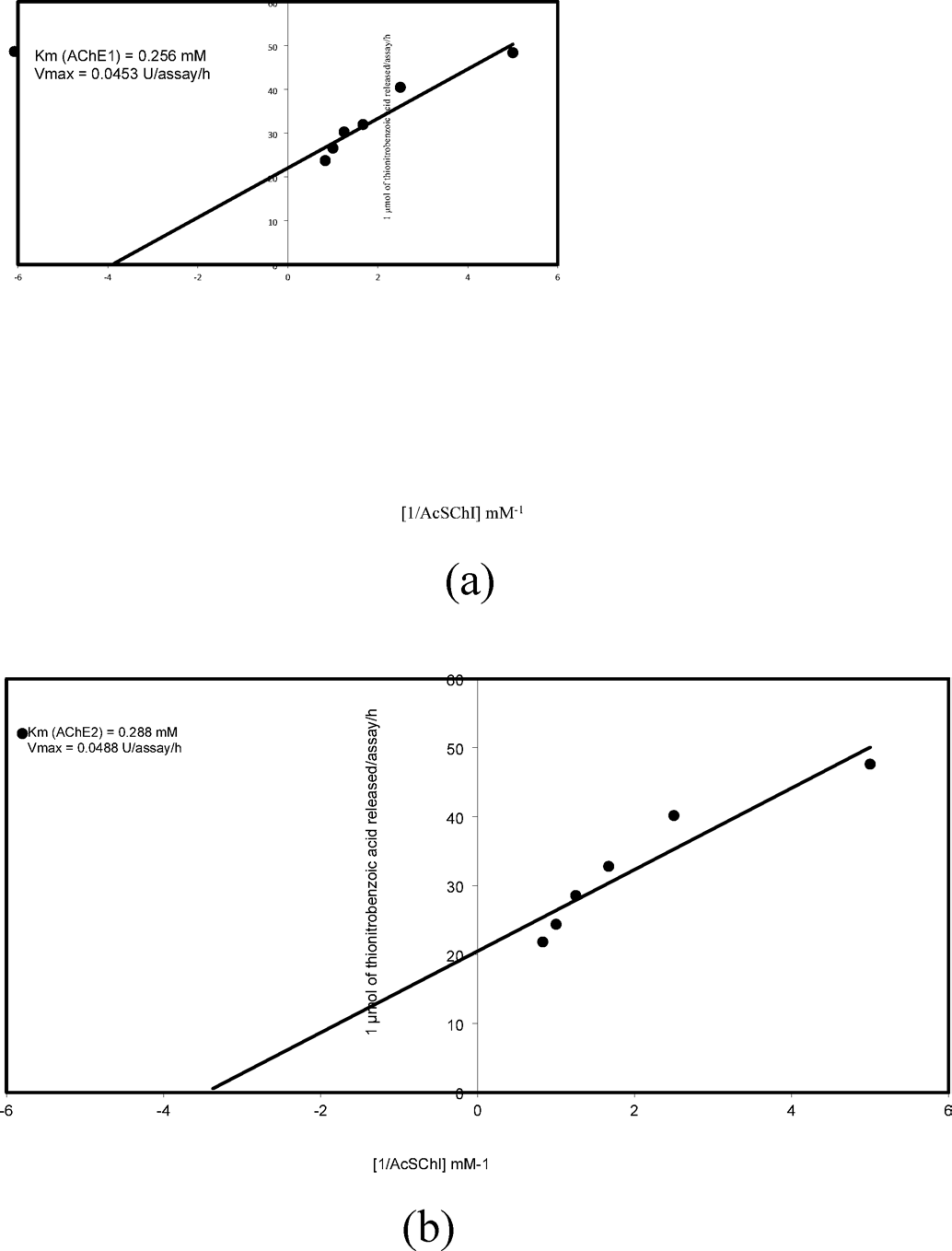
Lineweaver-Burk plot relating *Ae. aegypti* 36 h-old eggs AChE1 **(a)** and AChE2 **(b)** reaction velocity to AcSChI concentration. Each point represents the average of two experiments.

In this study, the expression AChE forms during developmental stages of *Ae. aegypti* were detected by using chromatography on a Sephacryl S-200 column. For all developmental stages two separate peaks of acetylcholinesterases AChE1 and AChE2 were observed (Fig. S1). The partial purification of acetylcholinesterase was summarized in Table 1. Different expression patterns of AChE1 and AChE2 activities were detected. The highest expression of AChE1 activity was detected at the egg (37.3%) and gradually decreased during larvae (26.9%), pupa (7.87%) and adult (5.13%). In contrast, the level of the activity of AChE2 was lowest at egg (17%), larvae (16.9%) and gradually increased at pupa (24.3%) and reached the highest level at adult (55.4%). Acetylcholine’s well-established function as a non-neuronal signaling molecule during early embryogenesis, where it helps regulate cell proliferation, differentiation, and morphogenetic processes before synapse formation, is discussed in relation to the predominance of AChE1 in eggs^27^. In accordance with findings in a number of insect and invertebrate models, it is believed that AChE activity at these early stages regulates basal cholinergic signaling rather than classical neurotransmission^22^. On the other hand, it is thought that AChE2’s fundamental function in synaptic transmission within mature neuronal and neuromuscular systems is reflected in its peak expression and activity in adults. In mature insects, where quick acetylcholine hydrolysis is necessary for movement, sensory processing, and reproductive activities, AChE2 has been extensively documented as the predominant synaptic isoform^28^.

**Table 1:** Partial purification scheme for *Ae. aegypti* acetylcholinesterase (AChE) during development.

Sample	Total activity (Units) *	Total protein (mg)	Specific activity	Purification fold	% Recovery
			(Units/mg protein)		
36h-old eggs					
crude extract	10.9	0.63	17.3	1.00	100
Chromatography on Sephacryl S-200					
AChE1	4.07	0.58	7.01	0.40	37.3
AChE1	1.86	0.16	11.6	0.67	17.0
Second-instar larvae					
crude extract	10.5	0.85	12.3	1.00	100
Chromatography on Sephacryl S-200					
AChE1	2.83	0.32	8.84	0.72	26.9
AChE1	1.78	0.13	13.6	1.10	16.9
Pupa					
crude extract	6.08	2.18	2.78	1.00	100
Chromatography on Sephacryl S-200					
AChE1	0.48	0.13	3.69	1.32	7.89
AChE1	1.48	0.15	9.87	3.55	24.3
Adult					
crude extract	11.1	2.10	5.29	1.00	100
Chromatography on Sephacryl S-200					
AChE1	0.57	0.10	5.70	1.07	5.13
AChE1	6.15	0.57	10.8	2.04	55.4

*One unit of AChE activity is defined as the amount of enzyme which released one µmol of thionitrobenzoic acid per h under standard assay conditions. Each value represents the average of two experiments.

AChE1 and AChE2 from 36 h-old *Ae. aegypti* eggs were physiochemically characterized. To be farther away from the larval stage, 36 h-old eggs were selected rather than 48-hour-old eggs. Acetylthicholine iodide (AcSChI) is considered to have 100% relative activity in terms of substrate specificity. In comparison to AcSChI, the relative activity percentage for butyrylthicholine iodide (BuSChI) was 9.4 for AChE1 and 11.6 for AChE2 (Table 2). AcSChI had the highest AChE activity in *P. pruinosus*^29^ and *H. bacteriophora*^30^, while the other substrates showed less activity. When AChEs from the mosquito Culex pipiens were characterized, it was found that AChE1 and AChE2 had different substrate specificities^31^.

**Table 2:** Relative activities of *Ae. aegypti* 36 h-old eggs AChE1 and AChE2 toward different substrates. Each value represents the average of two experiments.

Substrate	% Relative activity	
	AChE1	AChE2
AcSChI	100	100
BuSChI	9.40	11.6

The Km values of 0.253 and 0.288 mM were detected for *Ae. aegypti* 36 h-old eggs AChE1 and AChE2 with moderate affinity for AcSChI, respectively (Fig. 3). AChE from the nematoda *Heterohabditis bacteriophora* was shown to have a comparable Km (0.27 mM)^30^. AChEs from *Schizphis graminum* (Km 0.059 mM^32^) and *Bactrocera dorsalis* (Km 0.088^33^) were shown to have low Kms.

**Fig. 4:**
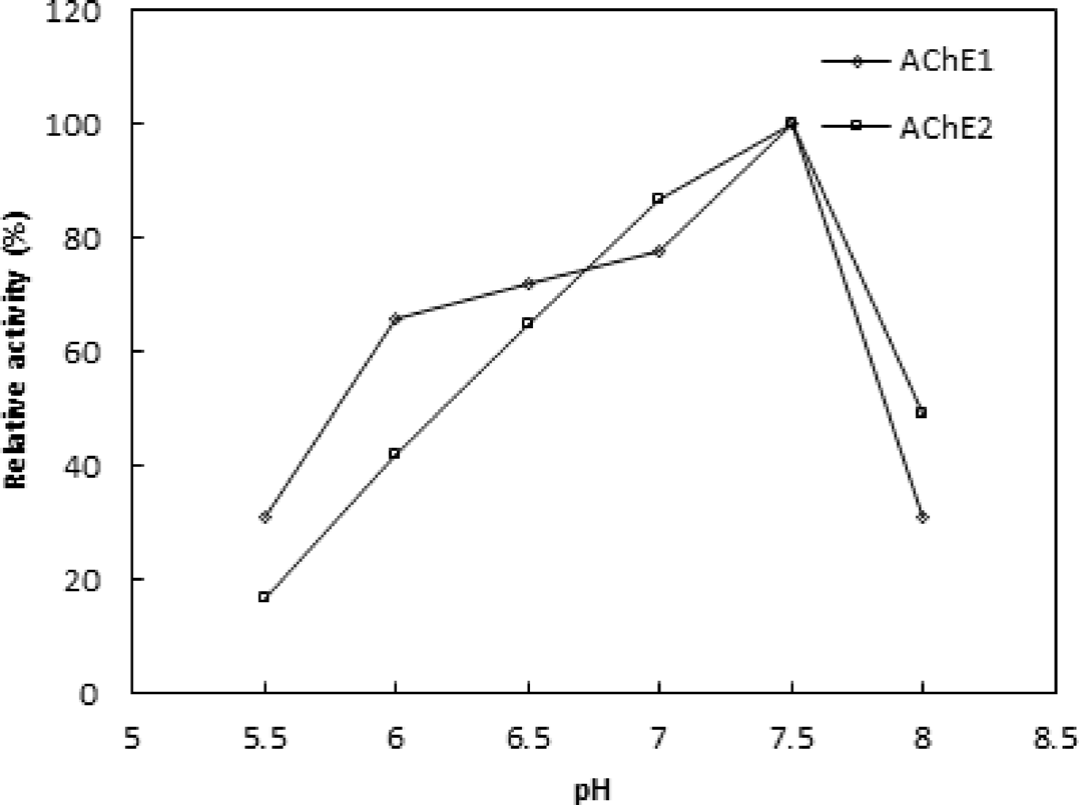
pH optimum of AChE1 and AChE2. Each point represents the average of two experiments.

The pH optimum of *Ae. aegypti* 36-hour-old eggs AChE1 and AChE2 was comparable at 7.5. However, AChE2 was found to have a broad pH range of 6.0 to 7.0 (Fig. 4). The isolated enzyme from *B. dorsalis*^33^ and H. bacteriophora^30^ showed a comparable pH optimum. AChE from Gammarus pulex^34^ and green rice leafhopper (*Nephotettix cincticeps*)^35^ were shown to have higher pH optima of 7.8 and 8.5, respectively.

**Fig. 5:**
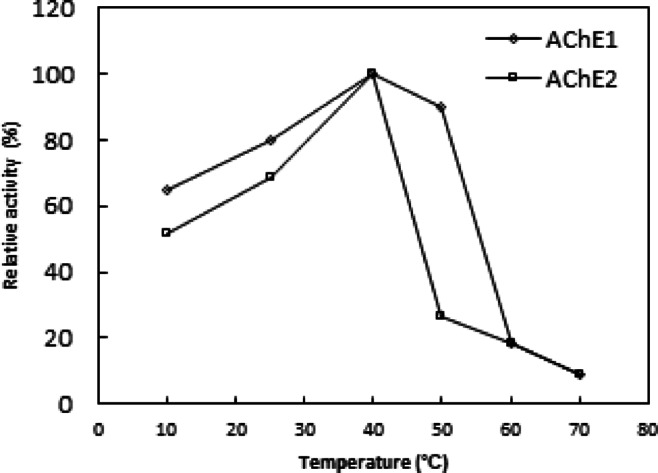
Temperature optimum of AChE1 and AChE2. Each point represents the average of two experiments.

**Fig. 6:**
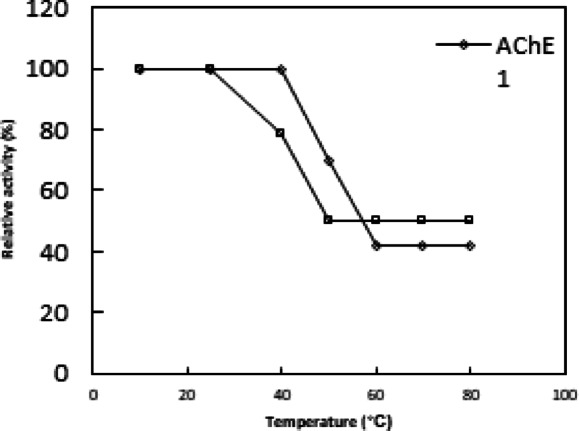
Effect of temperature on the thermal stability of AChE1 and AChE2. Each point represents the average of two experiments.

*Ae. aegypti* 36 h-old eggs AChE1 and AChE2 were found to have the same temperature optimum at 40˚C. At 50˚C, AChE2 lost 80% of its activity while AChE1 maintained 90% (Fig. 5). This temperature optimum matched the findings for the AChE from *H. bacteriophora*^30^ and *B. dorsalis*^33^. According to Li and Han^36^, AChE from *D. melanogaster* has a lower temperature optimum of 25 °C. *Ae. aegypti* 36-hour-old eggs AChE1 exhibited thermal instability above 50 °C, with 30 and 58% of its activity reduced after 30 min of incubation at 60 and 70 °C, respectively (Fig. 6). Similarly, *H. bacteriophora* AChE’s thermal stability was unstable above 50 °C; after 30 min of incubation at 60, 70, and 80 °C, respectively, 46, 63, and 80% of its activity was lost^30^. According to Joshi and Singh^37^, after five minutes of incubation at 60 °C, 60% of *H. contortus* AChE activity was eliminated. On the other hand, AChE2 for 36 h-old *Ae. aegypti* eggs exhibited instability above 25 °C, losing 21 and 50% of its activity after 30 min of incubation at 40 and 50 °C (Fig. 6). However, after being incubated for 10 and 20 min at 50 and 45 °C, respectively, *B. dorsalis* and *A. gossypii* AChEs lost 50 and 65% of their activity^33,34^.

36 h-old *Ae. aegypti* eggs were partially inhibited by all of the metal ions tested at 1 mM concentrations (Table 3). Pb^+ 2^, Cu^+ 2^, and Zn^2+^ displayed distinct inhibitory effects on both enzymes, but Mg^+ 2^, Ca^+ 2^, Hg^+ 2^, and Ni^+ 2^ had comparable inhibitory effects. Conversely, AChE1 and AChE2 were completely inhibited by Co^+ 2^. For AChE from *Arthrobacter ilici*, a comparable impact of Co^2+^ was found^38^. Cu^+ 2^, Ni^+ 2^, Hg^2+^, and Pb^+ 2^ partially inhibited AChE from *Clarias batrachus*^39^. AChEs from *Narke japonica* and *Torpedo californica* were shown to be activated by Ca^2+^ and Mg^+ 2^^40^, but AChE from *Arthrobacter ilicis* was unaffected^38^. Zn^2+^ stimulated adult *Ascaridia galli’s* AChE^41^.

**Table 3:** Effect of metal ions on *Ae. aegypti* 36 h-old eggs AChE1 and AChE2. Each value represents the average of two experiments.

Metal cation	Relative activity%	
	AChE1	AChE2
Mg^+ 2^	47	46
Pb^+ 2^	41	33
Ca^+ 2^	57	54
Cu^+ 2^	50	68
Co^+ 2^	0.0	0.0
Ni^+ 2^	47	47
Hg^+ 2^	71	72
Zn^+ 2^	27	48

From the above results, it can be concluded that the physiochemical properties of AChE1 and AChE2 from 36 h-old *Ae. aegypti* eggs were similar or different than that properties of other insects and nematode.

Because they block AChE, insecticidal active organophosphate and carbamate esters have been shown to be hazardous to insects. A wide range of chemical structures with various chemical and physical characteristics were used to represent the organphosphorus and carbamate insecticides^15^. For organophosphate or carbamate as inhibitors, the type of inhibition, bimolecular rate constant (Ki), and Ki ratio *Ae. aegypti* 36 h-old eggs AChE1/AChE2 were detected (Table 4). With the exception of eserine, which caused noncompetitive and competitive inhibition for AChE1 and AChE2, respectively, all inhibitors caused competitive inhibition for both enzymes. For AChE1 and AChE2, the lower Ki values of 0.45 and 0.33 µM fenitrothion were detected, respectively. AChE1 and AChE2 were shown to have moderate Ki values for eserine (5.9 and 1.37 µM, respectively). The insecticide with the highest sensitivity had a lower Ki value. Eserine was found to inhibit AChE of *H. bacteriophora* competitively, with a Ki value of 3.5 µM^30^. AChE from the brain tissues of *Clarias Batrachus* and *Oreochromis Mossambica* showed lower Ki values of 0.34 and 0.24 µM eserine, respectively^42^.

**Table 4:** Kinetic inhibition of *Ae. aegypti* 36 h-old eggs AChE1 and AChE2 by carbamates and organophosphates. Each value represents the average of two experiments.

Insecticides	Enzyme	Inhibition type	K_i_	K_i_ ratio
			(µM)	AChE1/AChE2
Methomyl	AChE1	Competitive	1.84	0.90
	AChE2	Competitive	2.04	
Eserine	AChE1	Non-competitive	5.90	4.30
	AChE2	Competitive	1.37	
Malathion	AChE1	Competitive	6.09	1.90
	AChE2	Competitive	3.20	
Chlorpyrifos-methyl	AChE1	Competitive	2.98	0.69
	AChE2	Competitive	4.30	
Fenitrothion	AChE1	Competitive	0.45	1.36
	AChE2	Competitive	0.33	
Pirimiphos-methyl	AChE1	Competitive	2.41	0.48
	AChE2	Competitive	4.97	

*Ae. aegypti* 36 h-old eggs are thought to be more sensitive to the inhibitor if their Ki ratio AChE1/AchE2 is less than 1.0, and vice versa (Table 4). According to the findings, the Ki ratio AChE1/AChE2 toward methomyl, chlorpyrifos-methyl, and pirimiphos-methyl is less than 1.0, suggesting that both enzymes are more susceptible to inhibition by these insecticides. Eserine, malathion, and fenitrothion, on the other hand, exhibited a Ki ratio of AChE1/AchE2 more than 1. Similar outcomes were noted for AChEs from *Bombyx mandarina* silkworms with Kis 1.14 and 0.45 µM eserine, respectively, with a Ki ratio of 2.53, and with Kis 1.28 and 0.12 µM methomyl, respectively, with a ratio of 10.66^43^.

The distinct activity and inhibition profiles of AChE1 and AChE2 observed in *Ae. aegypti* eggs suggest that the cholinergic system is functionally relevant even at early developmental stages. This highlights the egg stage as a potentially valuable but underutilized target in integrated vector management strategies. Targeting embryonic AChE activity through stage-specific insecticides could disrupt mosquito population development before larval emergence, thereby reducing overall vector abundance and reliance on later-stage chemical interventions. Incorporating egg-targeted control measures may also help distribute selection pressure across the life cycle, potentially slowing resistance development. However, while these findings indicate biochemical susceptibility at the egg stage, further field-based validation is required to confirm operational effectiveness.

However, stage-specific differences in AChE activity may not arise solely from normal developmental regulation linked to nervous system maturation. While changes in enzyme expression across life stages likely reflect shifts in physiological function—from embryonic signaling to synaptic neurotransmission—alternative explanations should also be considered. These variations may represent adaptive responses to environmental pressures, such as metabolic demands or potential exposure to toxins and insecticides. In this view, differential AChE activity could reflect stage-specific preparedness or tolerance mechanisms rather than purely ontogenetic programming. Considering both developmental regulation and adaptive responsiveness provides a more balanced understanding of why AChE activity varies across life stages and highlights the need for future work to distinguish intrinsic biological control from environmentally influenced modulation.

## Conclusion

In the present study, the cholinergic system is active at an early developmental stage, according to biochemical characterisation of AChE in *Ae. aegypti* eggs, making eggs a possible target for mosquito control. Using insecticides that act before larvae emerge can be guided by knowledge of stage-specific enzyme sensitivity. By applying pressure during several life phases, incorporating egg-targeted interventions into vector management techniques may increase control effectiveness, lessen dependency on later-stage treatments, and aid in resistance management. Under laboratory conditions, methomyl, pirimiphos-methyl, and chlorpyrifos-methyl showed significant acetylcholinesterase inhibition at the egg stage; nevertheless, these results should be interpreted cautiously. Because environmental variables, exposure dynamics, and population variability may affect results, enzyme inhibition tests carried out in controlled settings may not always translate into field-level efficacy. Therefore, before suggesting their widespread use in mosquito control programs, more field-based assessments are necessary.

## Supplementary Information

Below is the link to the electronic supplementary material.

Supplementary Material 1

## Data Availability

The datasets generated during and/or analyzed during the current study are available from the corresponding author upon reasonable request.
