# Correction: The patterns of acetylcholinesterases during developmental stages of *Aedes aegypti* and their susceptibility toward insecticides in egg stage

**DOI:** 10.1038/s41598-026-53190-3

**Published:** 2026-05-19

**Authors:** Saleh A. Mohamed, Azza M. Abdel-Aty, Hasan A. Al-Talhi, Ahmed N. Abo-Khatwa, Khaled M. Al-Ghamdi

**Affiliations:** 1 Molecular Biology Department, National Research Center, Dokki, Cairo, Egypt; 2 Biochemistry Department, Faculty of Science, King Abdulaziz University, 21589 Jeddah, Kingdom of Saudi Arabia; 3 Biology Department, Faculty of Science, King Abdulaziz University, 21589 Jeddah, Kingdom of Saudi Arabia

Correction to: *Scientific Reports* 10.1038/s41598-026-45818-1, published online 18 April 2026

The original version of this Article contained an error in the spelling of the author Azza M. Abdel-Aty which was incorrectly given as Azza A. Abdel-Aty.

The original Article has been corrected.

