# Supplementary figures and images for: The patterns of acetylcholinesterases during developmental stages of Aedes aegypti and their susceptibility toward insecticides in egg stage
Source: Sci Rep. 2026 Apr 18;16:12730. doi: 10.1038/s41598-026-45818-1 (PMC13091793; doi:10.1038/s41598-026-45818-1)

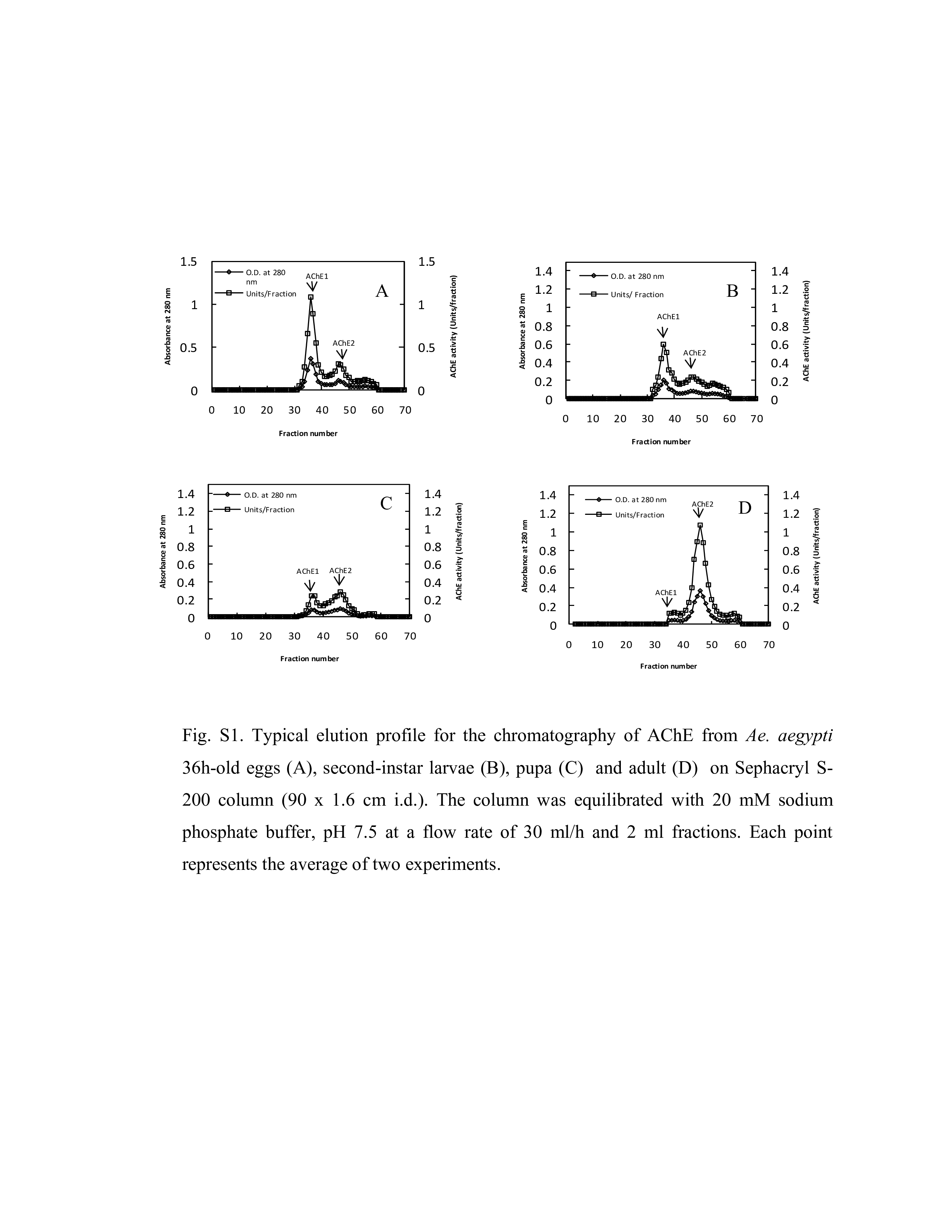

Supplement: Supplementary file 1 — Supplementary Material 1 [file 41598_2026_45818_MOESM1_ESM.jpg]
